# Generation of complement protein C3 deficient pigs by CRISPR/Cas9-mediated gene targeting

**DOI:** 10.1038/s41598-017-05400-2

**Published:** 2017-07-10

**Authors:** Wei Zhang, Guan Wang, Ying Wang, Yong Jin, Lihua Zhao, Qiang Xiong, Lining Zhang, Lisha Mou, Rongfeng Li, Haiyuan Yang, Yifan Dai

**Affiliations:** 1 Jiangsu Key Laboratory of Xenotransplantation, Nanjing Medical University, Nanjing, 211166 P.R. China; 2 Shenzhen Xenotransplantation Medical Engineering Research and Development Center, Institute of Translational Medicine, Shenzhen Second People’s Hospital, First Affiliated Hospital of Shenzhen University, Shenzhen, Guangdong 518035 China

## Abstract

Complement protein C3 is the pivotal component of the complement system. Previous studies have demonstrated that C3 has implications in various human diseases and exerts profound functions under certain conditions. However, the delineation of pathological and physiological roles of C3 has been hampered by the insufficiency of suitable animal models. In the present study, we applied the clustered regularly interspaced short palindromic repeat (CRISPR)/CRISPR-associated (Cas) system to target the *C3* gene in porcine fetal fibroblasts. Our results indicated that CRISPR/Cas9 targeting efficiency was as high as 84.7%, and the biallelic mutation efficiency reached at 45.7%. The biallelic modified colonies were used as donor for somatic cell nuclear transfer (SCNT) technology to generate C3 targeted piglets. A total of 19 *C3* knockout (KO) piglets were produced and their plasma C3 protein was undetectable by western blot analysis and ELISA. The hemolytic complement activity and complement-dependent cytotoxicity assay further confirmed that C3 was disrupted in these piglets. These *C3* KO pigs could be utilized as a valuable large animal model for the elucidation of the roles of C3.

## Introduction

The complement system is an important mechanism of innate immunity and plays an essential role in bacteriolysis, inflammation, opsonization, phagocytosis, and humoral and cellular immune responses^1, 2^. Three established pathways of complement activation exist, including the classical, alternative, and lectin pathways^3^. The activation events of all pathways converge at the formation of a convertase, which cleaves the central molecule of the cascade, namely, the third complement component (C3). C3 is the central component of the complement system and is encoded by the *C3* gene. Previous studies have demonstrated the importance of C3 in the adaptive immune response^4–6^. Although C3 is primarily synthesized by hepatocytes^4^, other tissues and cells also have the capability to synthesize C3 such as macrophages^7^, dendritic cells^8^, polymorphonuclear leukocytes^9^, proximal tubular epithelial cells^10–12^, fibroblasts^13^, uterine epithelia^14^, pneumonocytes^15^, and activated T cells^16^, indicating that it may possess pleiotropic physiological function. Besides its implications in various congenital and acquired complement deficiencies, C3 is involved in glomerular disease^17^, hemolytic uremic syndrome^18^, and gastric carcinoma^19^. Based on its pivotal roles in complement activation and multiple biological functions, researchers have been prompted to address both pathological and physiological activities of C3 using C3-deficient animal models.

To date, several inherited C3 deficiencies in humans^20^, guinea pigs^21^, dogs^22^, rabbits^23^, and *C3* knockout animal models^24^ have been reported and have provided insights into understanding the diversified biological responses mediated by C3. However, these animal models have limitations due to their physiology and gene expression, which differ from humans, and studies involving large C3-deficient animal models have not been conducted. Pigs have the advantage over rodent models in that they share more similarities with humans in terms of anatomy, physiology, immunology, and clinical relevance^25–27^. Pigs are also the preferred animal model because of its short gestation period and large litter size. Moreover, compared to other large animals such as non-human primates, pigs are more ethically and economically acceptable. Currently, pigs are extensively used as a large-animal model in biomedical research^28^.

Recently, the clustered regularly interspaced short palindromic repeats/CRISPR-associated protein 9 (CRISPR/Cas9) system has significantly improved gene targeting efficiency and has been extensively used to generate genetically modified animal models in various species, including mice^29^, rabbits^30^, sheep^31^ and pigs^32^. In the present study, we successfully generated *C3* targeted piglets by using the CRISPR/Cas9 system combined with the somatic cell nuclear transfer (SCNT) technology. These *C3* KO pigs may serve as valuable large-animal models for further delineation of various functional roles played by C3.

## Results

### CRISPR/Cas9-mediated gene targeting of *C3* in pig primary fetal fibroblast (PFFs)

**Figure 1:**
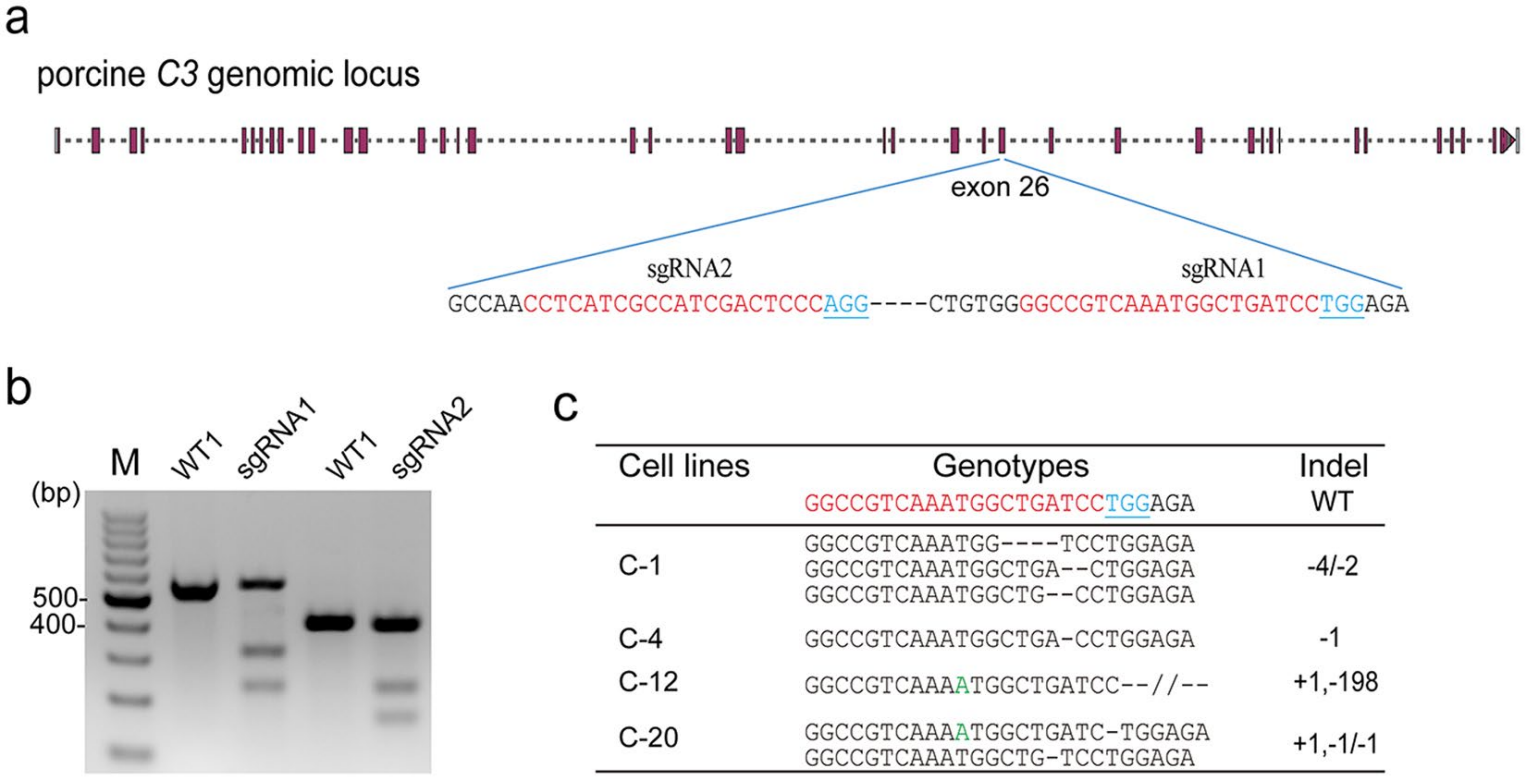
CRISPR/Cas9-mediated C3 gene targeting in PFFs. (**a**) Schematic diagram of sgRNA targeting the exon 26 of the *C3* gene. sgRNA targeting sites are highlighted in red. PAM sequences are underlined and highlighted in blue. (**b**) T7E1 assay for CRISPR/Cas9-mediated cleavage at target site in PFFs. WT1 and WT2: PCR products of untransfected PFFs treated with T7E1; sgRNA1 and sgRNA2: PCR products of PFFs transfected with Cas9-sgRNA1 and Cas9-sgRNA2 treated with T7E1. (**c**) Biallelic mutation donor cell sequences for SCNT. The wild-type sequence is shown at the top of the target sequence. The sgRNA sequences are highlighted in red, PAM sequences in blue, and insertions in green; deletion (−); insertion (+).

To disrupt the function of the *C3* gene in pigs, two sgRNAs targeting the exon 26 were designed using online tools (http://crispr.mit.edu/). The target sites are shown in Fig. 1a. To test their cleavage efficiency, these two sgRNAs were cloned into pX330 vector and transfected into PFFs derived from Bama mini pigs. Genomic DNA was isolated from PFFs after 48 h transfection. PCR amplicons spanning the *C3* target region were subjected to T7E1 assay analysis. The results showed that both sgRNAs could target the *C3* gene, and the mutation efficiency of sgRNA1 and sgRNA2 was 53.2% and 39.7%, respectively (Fig. 1b). To establish *C3* knockout (KO) cell lines, the Cas9-sgRNA1 targeting vector was co-transfected with a TD-tomato plasmid containing a neomycin resistance (*Neo*) gene into an early passage of primary PFFs that were derived from a 35-day-old male Bama mini fetus. A total of 46 G418-resistant colonies were isolated after G418 selection for 10 days. Genotyping analysis of each colony was performed using TA-cloning and Sanger sequencing. The results indicated that 21 out of the 46 colonies harbored a homozygous/heterozygous biallelic mutation in the *C3* gene target region (Table 1).

**Table 1:** *C3* targeting in PFFs using the CRISPR/Cas9 system.

Monoallelic-KO	Biallelic-KO	Indel-positive
18/46 (39.1%)	21/46 (45.7%)	39/46 (84.7%)

### Generation of *C3* KO pigs via SCNT

**Figure 2:**
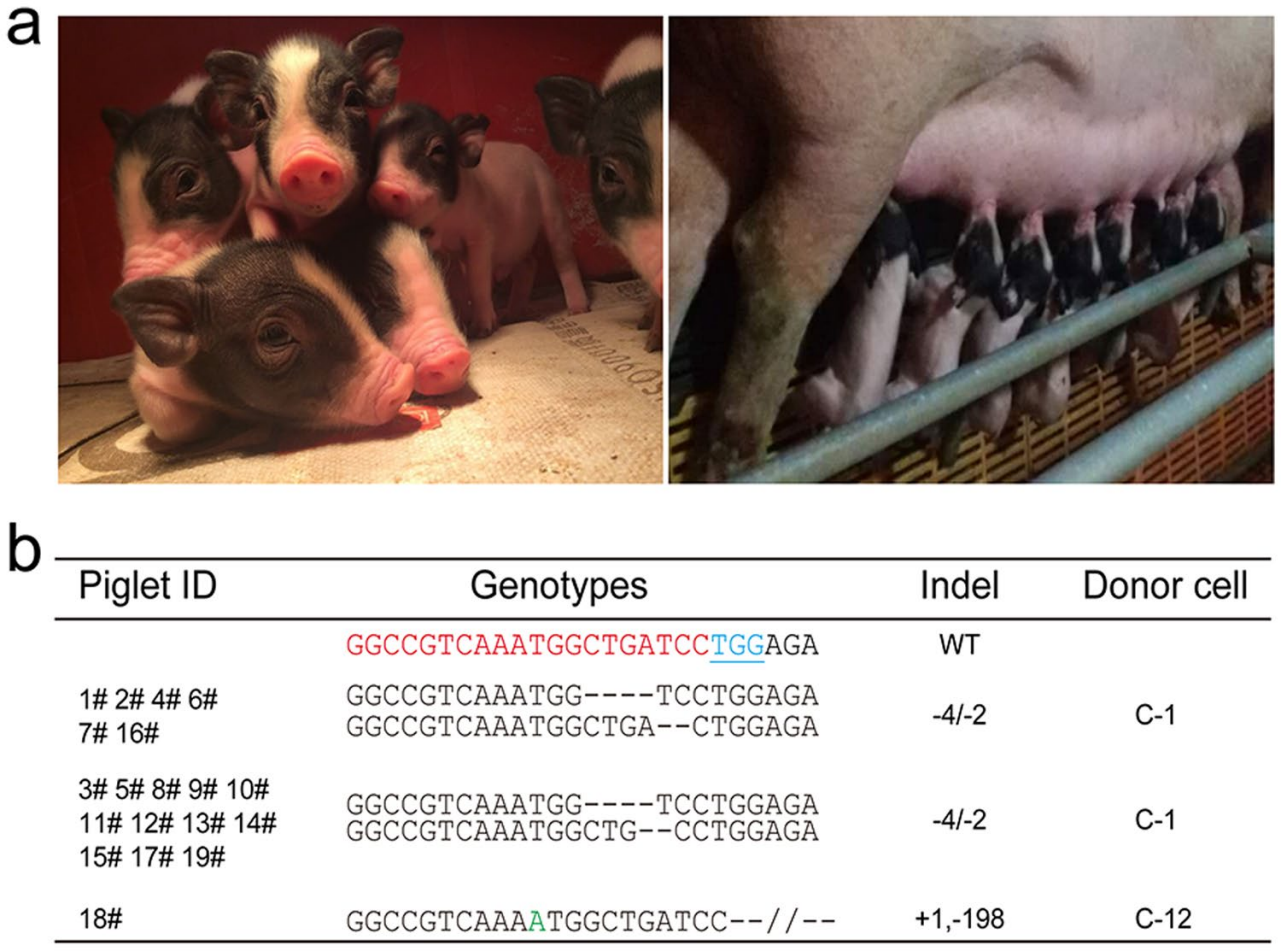
Generation of *C3* mutant piglets via SCNT. (**a**) Photograph of newborn cloned piglets carrying *C3* gene mutation. (**b**) *C3* genotypes of cloned piglets. The wild-type (WT) sequence is shown at the top in which the sgRNA sequences are highlighted in red, PAM sequences in blue, and insertions in green. WT: wild type; deletion (−); insertion (+).

To generate *C3* KO piglets, C-1, C-4, C-12, and C-20 biallelic modified colonies were used as donor cells for SCNT (Fig. 1c). A total of 3,168 reconstructed embryos were transferred into 9 surrogates. B-ultrasonic examination was performed on each recipient about 30 days after surgery, and 3 of the 9 gilts were found to be pregnant, indicating a pregnancy rate of 33.3% (Table 2). The 3 recipients were maintained to term and gave birth to 19 live-born piglets (Fig. 2a). The genotype of each clone piglet was determined by TA-cloning and sequencing using DNA isolated from ear tissues. All of the 19 piglets were confirmed as biallelic *C3* mutants. Notably, only 1 of the 19 piglets carrying a +1/−198 bp mutation was derived from C-12 donor cells, and the remaining piglets derived from C-1 donor cells were grouped into two types of −4/−2 bp mutations indicating that C-1 was a mixture of cell colonies (Fig. 2b and Table 3). To examine whether the transfected plasmids integrated into the cloned piglet genomes, PCR amplification was performed using genomic DNA from cloned piglets by primers specifically for Cas9 and *Neo* gene. Our results showed that there was no integration of the Cas9-sgRNA1 vector or the TD-tomato plasmid into these piglet genomes (see Supplementary Fig. S1). All piglets were healthy before weaning from their mother. However, some piglets became sick due to infection or diarrhea after weaning and were euthanized at 2–3 months of age.

**Table 2:** Somatic cell nuclear transfer results for the generation of *C3* knockout cloned pigs.

Cell pool	Number of transferred embryos	Number of recipients	Number of pregnancies	Number of piglets born	Number of mutants
C-1, C-12, C-20	348	1	0	—	—
C-1, C-12, C-20	335	1	0	—	—
C-1, C-12, C-20	356	1	1	6	6
C-1, C-12, C-20	375	1	0	—	—
C-1, C-12, C-20	375	1	0	—	—
C-1, C-12, C-20	386	1	0	—	—
C-1, C-4, C-12, C-20	320	1	0	—	—
C-1, C-4, C-12, C-20	323	1	1	10	10
C-1, C-4, C-12, C-20	325	1	1	3	3
Total	3143	9	3 (33%)	19	19

**Table 3:** Details on the generated *C3* KO piglets.

Piglets	Genotypes	Indel	Birth weight (kg)	Donor cell
	AG	wt		
1#	GGCCGTCAAATGG----TCCTGGAG	−4/−2	0.5	C-1
	GGCCGTCAAATGGCTGA--CTGGAG			
2#	GGCCGTCAAATGG----TCCTGGAG	−4/−2	0.85	C-1
	GGCCGTCAAATGGCTGA--CTGGAG			
3#	GGCCGTCAAATGG----TCCTGGAG	−4/−2	0.80	C-1
	GGCCGTCAAATGGCTG--CCTGGAG			
4#	GGCCGTCAAATGG----TCCTGGAG	−4/−2	1.20	C-1
	GGCCGTCAAATGGCTGA--CTGGAG			
5#	GGCCGTCAAATGG----TCCTGGAG	−4/−2	0.95	C-1
	GGCCGTCAAATGGCTG--CCTGGAG			
6#	GGCCGTCAAATGG----TCCTGGAG	−4/−2	1.05	C-1
	GGCCGTCAAATGGCTGA--CTGGAG			
7#	GGCCGTCAAATGG----TCCTGGAG	−4/−2	0.94	C-1
	GGCCGTCAAATGGCTGA--CTGGAG			
8#	GGCCGTCAAATGG----TCCTGGAG	−4/−2	1.08	C-1
	GGCCGTCAAATGGCTG--CCTGGAG			
9#	GGCCGTCAAATGG----TCCTGGAG	−4/−2	0.705	C-1
	GGCCGTCAAATGGCTG--CCTGGAG			
10#	GGCCGTCAAATGG----TCCTGGAG	−4/−2	0.804	C-1
	GGCCGTCAAATGGCTG--CCTGGAG			
11#	GGCCGTCAAATGG----TCCTGGAG	−4/−2	0.979	C-1
	GGCCGTCAAATGGCTG--CCTGGAG			
12#	GGCCGTCAAATGG----TCCTGGAG	−4/−2	0.938	C-1
	GGCCGTCAAATGGCTG--CCTGGAG			
13#	GGCCGTCAAATGG----TCCTGGAG	−4/−2	1.067	C-1
	GGCCGTCAAATGGCTG--CCTGGAG			
14#	GGCCGTCAAATGG----TCCTGGAG	−4/−2	0.970	C-1
	GGCCGTCAAATGGCTG--CCTGGAG			
15#	GGCCGTCAAATGG----TCCTGGAG	−4/−2	0.605	C-1
	GGCCGTCAAATGGCTG--CCTGGAG			
16#	GGCCGTCAAATGG----TCCTGGAG	−4/−2	0.450	C-1
	GGCCGTCAAATGGCTG--CCTGGAG			
17#	GGCCGTCAAATGG----TCCTGGAG	−4/−2	0.360	C-1
	GGCCGTCAAATGGCTG--CCTGGAG			
18#	GGCCGTCAAATGGCTGATCC----//--	+1, −198	0.577	C-12
19#	GGCCGTCAAATGG----TCCTGGAG	−4/−2	0.724	C-1
	GGCCGTCAAATGGCTG--CCTGGAG			

sgRNA sequences are highlighted in red, PAM sequences in blue, and insertions in green. WT: wild type; deletion (−); insertion: (+).

### Characterization of *C3* KO piglets

**Figure 3:**
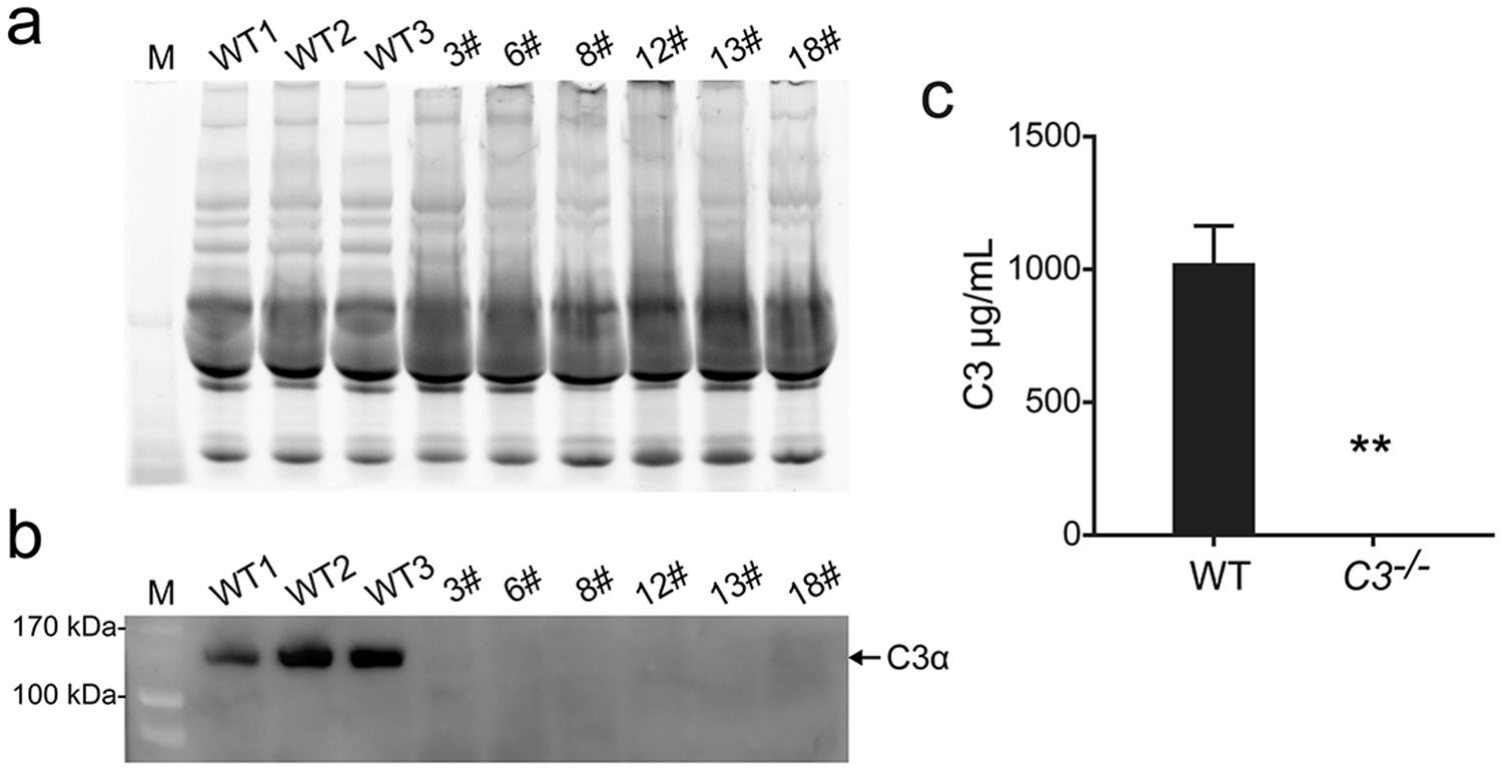
C3 protein expression analysis of piglet serum. (**a**) Stain-free gel image of total loaded serum proteins. (**b**) Western blot of serum C3 protein of cloned piglets compared to that of wild type piglets. Full-length blot is presented in Supplementary Figure S3. (**c**) Serum C3 protein levels of *C3* KO piglets and wild type controls were assessed by ELISA (**P < 0.01, data are shown as mean ± SEM, n = 3).

**Figure 4:**
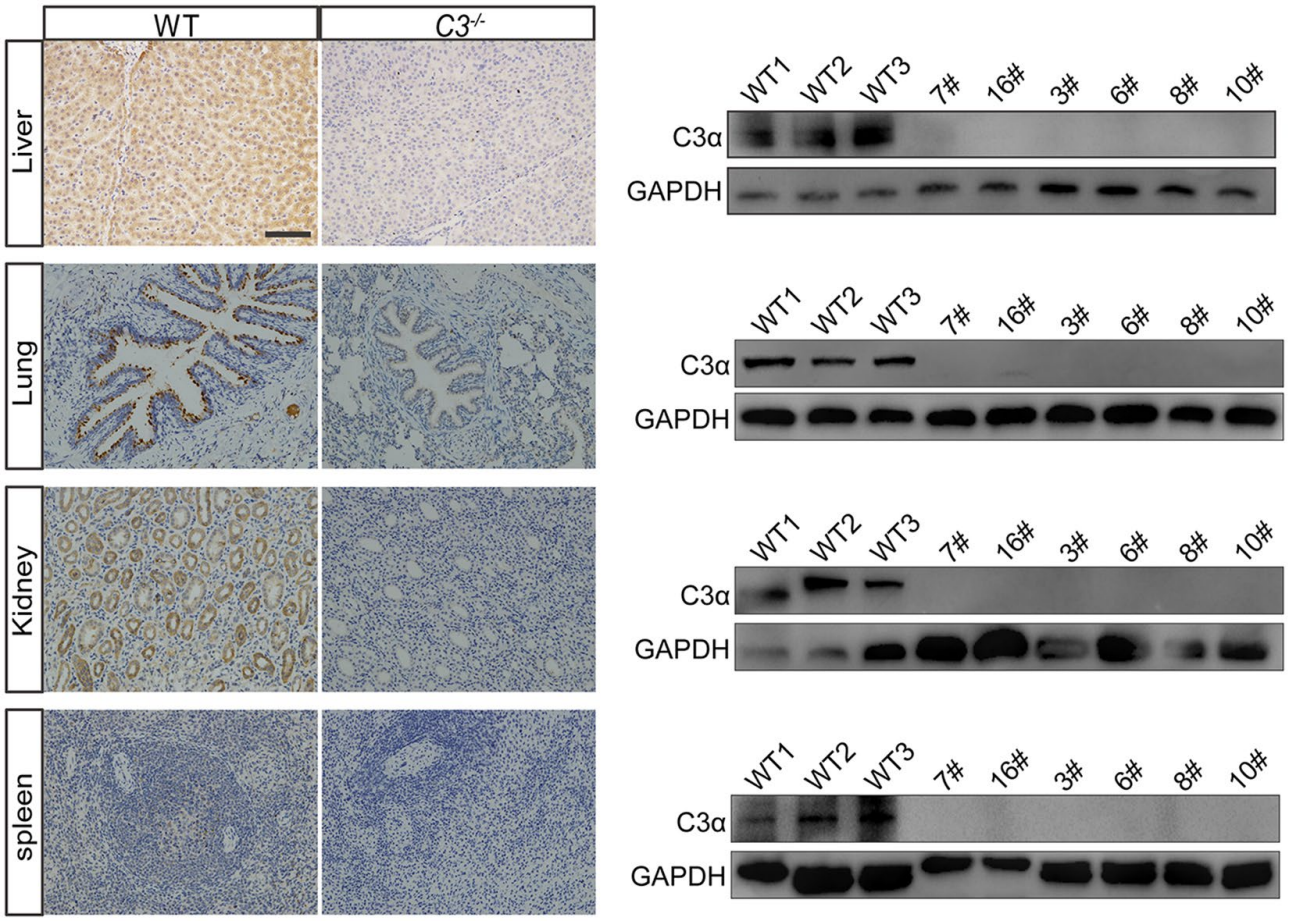
Immunohistochemistry (left) and western blot (right) analysis of C3 protein in various tissues. C3 protein was not detectable in liver, lung, kidney, and spleen of *C3* KO piglets. Full-length blots are presented in Supplementary Figures S4–S7. The scale bar is 100 μm.

To determine the C3 protein expression level of the gene targeted piglets, western blot was performed on fasting serum sampled from *C3* KO and age-matched wild-type (WT) pigs. Figure 3b showed that the C3 α-chain was undetectable in KO piglets contrary to that of the WT controls. Consistent with the results of western blot analysis, sandwich ELISA showed that the serum level of C3 protein was negative in the cloned piglets while the WT pigs had about 1,000 μg/mL of C3 protein (Fig. 3c). To further investigate the levels of C3 expression in different organs, including liver, kidney, lung, and spleen, immunohistochemistry was conducted on tissue sections. In contrast to the WT controls, no C3 signal was observed in any of the tissues of the *C3* KO piglets (Fig. 4, left) which was consistent with the western blot results (Fig. 4, right). Taken together, these results indicated that C3 expression in the *C3* KO piglets was completely disrupted by the CRISPR/Cas9 system.

### No complement activity was detected in the serum of *C3* KO piglets

**Figure 5:**
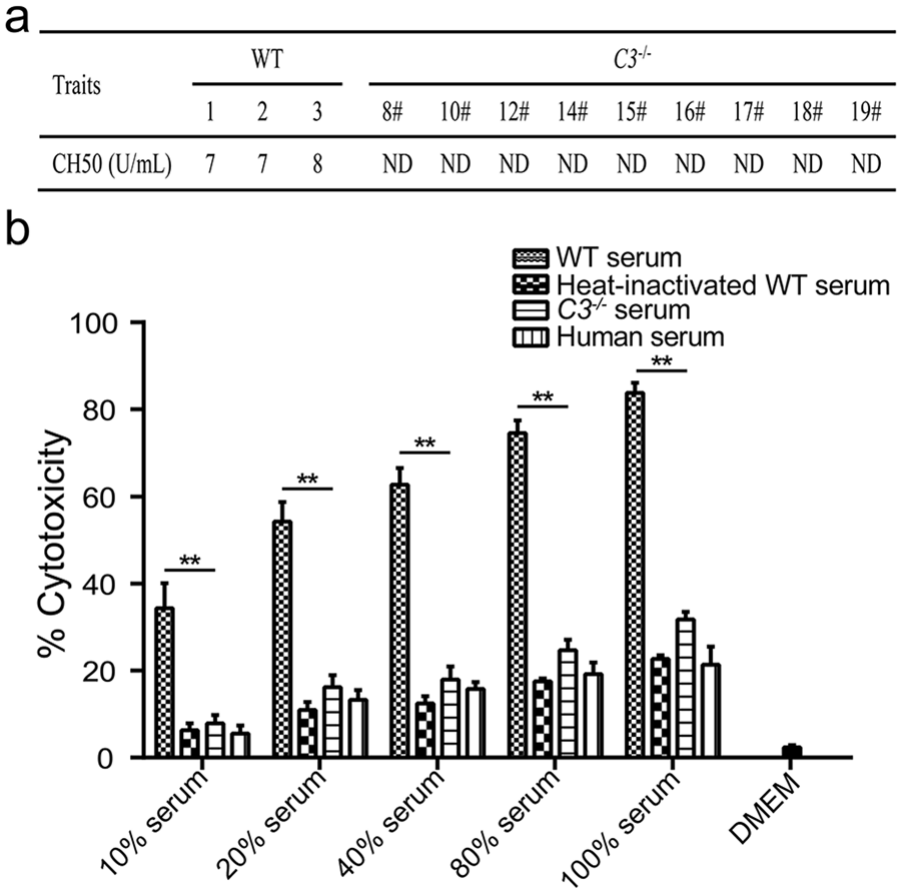
Hemolytic complement activity of pig serum and effects on anti-human cells. (**a**) Total hemolytic complement activities were determined by Dian Diagnostics. (**b**) The percentages of 293T cell lysis mediated by various concentrations of WT pig serum, heat-inactivated WT serum, *C3* KO piglet serum, and human serum. Lysis of 293T cells was significantly reduced when incubated in serum of *C3* KO piglets compared to WT controls. (**P < 0.01, data are shown as mean ± SEM, n = 5). ND: Not detectable.

To further confirm the *C3* KO piglets lack the complement activity, the complement hemolysis 50% assay (CH50) was performed by using a liposome immunoassay technique. It showed that the CH50 of the *C3* KO piglets was not detectable compared to that of the WT piglets (7.33 ± 0.33 U/mL) (Fig. 5a), indicating there is no complement activity in *C3* KO piglet serum. Serum cytotoxicity was tested with human 293T cells by using 10%, 20%, 40%, 80% and 100% of WT pig serum, heat-inactivated WT pig serum, *C3* KO piglet serum and human serum. Incubation with DMEM alone displayed only background cytotoxic activity for 293T (1.13%). WT serum showed a very potent cytotoxic effect on 293T which was enhanced with serum concentration increased (Fig. 5b). At the same concentrations, *C3* KO piglet serum showed comparable levels of cytotoxicity with heat-inactivated WT serum and human serum, which was significantly lower than that of WT serum, indicating complement activity in the *C3* KO piglet serum was dramatically reduced.

### Off-target analysis

Although the CRISPR/Cas9 system has the advantage of high gene targeting efficiency in pigs, the undesired off-target effects are still a major concern^33, 34^. To test whether off-target mutagenesis occurred in the *C3* KO piglets, 27 potential off-target sites (OTSs) for sgRNA1 were identified by screening the pig genomic DNA sequence. All of the 27 OTSs were PCR amplified using genomic DNA isolated from *C3* KO piglets and WT controls. The primers for amplifying the off-target sites are listed in Supplementary Table S1. These PCR products were sequenced and aligned. Sequencing chromatography of the PCR products spanning OTS16 region from *C3* KO piglets showed overlapped peaks, indicating that undesired DNA mutagenesis in OTS16 site was induced by sgRNA1 (see Supplementary Fig. S2). However, this mutagenesis was in a non-coding region of the pig genome, which was deemed to have no effect on *C3* deficiency.

## Discussion

In the present study, *C3*-deficient piglets were successfully generated by using the CRISPR/Cas9 system combined with SCNT technology. Our results demonstrated that there was no C3 protein in the plasma and tissues of the *C3* KO piglets (Figs 3 and 4); The complement-dependent cytotoxicity assay confirmed that lack of *C3* completely disrupted the activation of complement pathway in the *C3* KO piglets. Thus, we have successfully established a *C3*-deficient pig model which will be very useful to study many *C3* related diseases and phenotypes. Unfortunately, we have also found that lack of *C3* may cause some immune deficiency in the *C3* KO piglets and make them more susceptible to bacterial and viral infections. Therefore, a special pathogen free environment is necessary to keep them healthy before we can use them as large animal models in the future. Niemann has proposed that aberrant epigenetic events of cloned animals produced by the SCNT procedure may affect their phenotypes^35^. Currently, these *C3* KO founder pigs could not be maintained to reach their sexual maturity to produce F1 progeny. Therefore, a potential involvement of epigenetic status on the phenotype of *C3*-deficient cloned pigs cannot be excluded based on the data presented in this study. We should be able to determine the effect of epigenetic status once we are able to produce F1 offspring in a SPF environment.

Compared to zinc finger nucleases (ZFNs) and transcription activator-like effector nucleases (TALENs), CRISPR/Cas9 has been demonstrated to be more efficient and multiplexable in gene targeting^36^. In the present study, the *C3* gene targeting efficiency in PFFs that was mediated by CRISPR/Cas9 was as high as 53.2%, and the biallelic mutagenesis ratio reached 45.7% (Fig. 1b and Table 1). Thus, the high targeting efficiency and ease of use of the CRISPR/Cas9 gene-editing tool will greatly facilitate the production of gene KO pig models.

Instead of employing co-injection of Cas9 mRNA and sgRNA into one-cell stage embryos to generate gene-modified animals, we used eukaryotic plasmids expressing CRISPR/Cas9 to target the genome of PFFs. We used the clonability of specific mutant cells as donor nuclei and transferred the mixed embryos derived from different mutant cell lines to recipients to generate gene-targeting pigs (Table 2). The nucleofection of CRISPR/Cas9 plasmids into PFF nuclei may lead to higher targeting efficiency by enhancing the expression of the components of the CRISPR/Cas9 system. Furthermore, this strategy also avoids embryo toxicity and induction of non-determinacy of the genotypes from cloned piglets^37, 38^.

Off-target effects are a major concern of the CRISPR/Cas9 gene targeting system^33, 34, 39–41^. We utilized the CRISPR/Cas9 system and SCNT to generate 19 *C3*-deficient piglets in the present study, and only one off-target mutagenesis was detected in all of the 27 analyzed candidate OTSs in these animals. However, we are aware that off-target effects may exist in our cloned piglets as the *Sus scrofa* genome sequence is incomplete, which has hampered comprehensive searches for off-targets. Interestingly, we also found that almost all of the piglets were derived from a mixed KO cell line (C-1), except for one piglet (18#). The results indicated that the C-1 cell colony has higher competency or other cell colonies may contain undesired off-target mutations that result in embryonic lethality.

## Materials and Methods

### Ethics statement

This study was carried out in accordance with the guidelines approved by the Institutional Animal Care and Use Committee (IACUC) of the Nanjing Medical University, China. Tibetan mini pigs were housed in a large animal facility affiliated with Nanjing Medical University. All animals were fed chow diets purchased from Chia Tai (Jiangsu Huaiyin) Co., Ltd. twice a day. Standard pig husbandry procedures were applied to all animals.

### Plasmid construction

The pX330 plasmid (Addgene plasmid 423230) expresses human codon-optimized Cas9 and sgRNA under the chicken beta-actin hybrid (CBh) and human U6 promoters, respectively. Twenty-nucleotide sequences followed by the PAM sequence were designed as sgRNA (sgRNA1: GGCCGTCAAATGGCTGATCC; sgRNA2: CCTCATCGCCATCGACTCCC). The complementary oligos DNA of sgRNAs were synthesized and phosphorylated. The pX330 was digested with BbsI (Thermo Fisher Scientific, Waltham, USA) and ligated with the annealed oligos (37 °C for 30 min, 95 °C for 5 min, and annealed by decreasing 5 °C/min to 25 °C) to generate the Cas9-sgRNA targeting plasmids for *C3*.

### PFF isolation, culture, transfection, and screening

Primary porcine fetal fibroblast cells were isolated from the skin of fetuses at day 35 of gestation using 200 U/mL collagenase (Invitrogen, Carlsbad, USA) and 25 kU/mL DNaseI (Invitrogen, Carlsbad, USA) as previously described^42^. The PFFs were cultured in DMEM (Gibco, Grand Island, USA) medium containing 15% FBS (Gibco, Grand Island, USA) and 1× penicillin/streptomycin (Gibco, Grand Island, USA) at 37 °C in 5% CO2. Approximately 5 μg of the C3-Cas9-sgRNA plasmid and 1 μg of the neomycin-expression plasmid (pCMV-tdTomato) were transfected into 1 × 10^6^ PFFs by using the basic fibroblast nucleofection kit (Amaxa Biosystems/Lonza, Cologne, Germany) and nucleofection program U-023. Post-transfection cells were seeded into 10-cm dishes, and 48 h later, 800 μg/mL of G418 (Gibco, Grand Island, USA) was added to select single colonies for 10–14 days. The single colonies were isolated in 24-well plates and then passaged to 12-well plates. Approximately 1/5 of the cells of a single colony were lysed with NP-40 (55 °C for 30 min, 95 °C for 10 min) for PCR screening and 4/5 of the remaining cells were used for SCNT. The primers used in amplifying the target region were as follows: Forward: 5′-CCTCAGGTGGTCTAGGTTGG-3′, reverse: 5′-TTTTGCAATGGAGGAGTGGC-3′. The PCR conditions were as follows: 95 °C for 5 min, followed by 30 cycles of 95 °C for 10 s, 60 °C for 30 s, and 72 °C for 40 s, and a final 72 °C for 7 min. The PCR products were sequenced and then subcloned into a pMD18-T vector (Takara Clontech, Tokyo, Japan) according to the manufacturer’s instructions. Fifteen to twenty individual clones were picked and sequenced.

### SCNT and embryo transfer

The oocytes for SCNT were collected from ovaries purchased from a local slaughterhouse and cultured for 42–44 h *in vitro*. The mature oocytes were enucleated as described elsewhere^43^. A single targeted PFF cell was transferred into the perivitelline space of the enucleated oocyte. Subsequently, the donor cell and recipient cytoplast were fused and activated to form a reconstructed oocyte. Then, the reconstructed oocytes were cultured in an embryo-development medium for 4 h at 38.5 °C. About 300 reconstructed oocytes were transferred into the uterus of an estrus-synchronized recipient gilt. Pregnancy was confirmed by ultrasound 30 days after transplantation and monitored until perinatal period. All of the piglets were born by normal delivery.

### T7E1 cleavage assay

Fibroblasts transfected with or without Cas9-sgRNA plasmids (as earlier described) were cultured for 48 h. Genomic DNA was extracted using a DNA extraction kit (TianGen, Beijing, China) and the genomic region surrounding the CRISPR/Cas9 target site was PCR amplified. For sgRNA1, the forward primer was 5′-CCTCAGGTGGTCTAGGTTGG-3′, and reverse was 5′-TTTTGCAATGGAGGAGTGGC-3′. For sgRNA2, the forward primer was 5′-CGCCTCTCTCTGTTCATTGC-3′, and reverse was 5′-TTTTGCAATGGAGGAGTGGC-3′. The PCR conditions were as follows: 95 °C for 5 min, followed by 30 cycles of 95 °C for 10 s, 60 °C for 30 s, and 72 °C for 40 s, and a final 72 °C for 7 min. The T7E1 (NEB, Beverly, USA) cleavage assay was performed as described by Shen *et al*.^39^. Briefly, a total of 250 ng of the purified PCR product was mixed with NEB Buffer 2, denatured, and annealed to allow formation of heteroduplex using the following conditions: 95 °C for 5 min, 95 °C to 85 °C ramping at −2 °C/s, 85 °C to 25 °C at −0.1 °C/s, and 4 °C hold. After reannealing, the products were digested with 1 μL of T7 endonuclease I at 37 °C for 15 min and then run on a 2% agarose gel stained with ethidium bromide.

### Serum collection

To harvest serum, blood was collected by cardiac puncture and allowed to clot for 60 min at RT, followed by centrifugation at 3,000 rcf for 15 min at RT. The sera were stored at −80 °C.

### Complement-dependent cytotoxicity assay

The assay was conducted as previously described^44, 45^ with modifications. The human 293 T cells were diluted to 6 × 10^4^ cells per well in 96-well round-bottom plates. All cells were performed in DMEM medium containing 15% FBS and 1× penicillin/streptomycin. The cells were cultured for 24–48 h at 37 °C in 5% CO2. The liquid from each well was aspirated and incubated sequential dilution (1:1 to 1:10) of WT serum, heat-inactivated WT serum, *C3* KO piglet serum and human serum (Sigma, St.Lous, MO, USA) for 2 h at 37 °C, respectively. Heat-inactivated WT pig serum was achieved by a treatment at 56 °C for 30 min. DMEM were used as a negative control. After incubation, the cells were further incubated with 10% cell counting kit-8 (CCK8) (Dojindo, Kumamoto, Japan) for 2 h at 37 °C, which was followed by absorbance reading at a wavelength of 450 nm on a Safire2 TECAN plate reader. Cell killing was calculated as follows: Percent cytotoxicity = [(A − B)/(A − C)] × 100%, where A is the OD value of control (cells were cultured in fetal bovine serum), B represents the experimental group, and C is the blank (no cells, 10% CCK8 only). All assays were performed in duplicate.

### Western blot analysis

Western blot was used to detect C3 protein in pig serum. Approximately 5 μL of piglet serum was separated on 10% SDS-PAGE gels and transferred to polyvinylidene difluoride (PVDF) membranes (Millipore, Billerica, USA). The PVDF membranes were blocked with 5% non-fat powdered milk for 1 h at room temperature, incubated with anti-pig C3 α-chain antibody (Proteintech, Chicago, USA) at 4 °C overnight, and then rinsed with Tris-buffered saline (10 mM Tris-HCl, 150 mM NaCl, pH 7.5) and 0.05% Tween-20. Subsequently, the blots were incubated with a goat anti-rabbit IgG HRP-conjugated secondary antibody (1:5,000, Santa Cruz Biotechnology, Santa Cruz, USA). After washing three times, SuperSignal West Pico Chemiluminescent Substrate (Thermo Fisher Scientific, Waltham, USA) was used to develop the immunoblots.

### ELISA

A sandwich ELISA kit (LifeSpan BioSciences, Seattle, USA) was used to measure complement C3 in pig serum following the manufacturer’s recommendations, and then the plates were incubated with Sample for 90 min at 37 °C. The liquid from each well was aspirated and incubated with 1× a biotinylated detection antibody for 1 h at 37 °C. The plates were then washed three times with washing buffer, then an HRP conjugate and substrate solution were used for enzymatic detection. The reaction was terminated with 2NH_2_SO_4_ (aq) and absorbance was read at a wavelength of 450 nm, with a reference wavelength of 450 nm on a Safire2 TECAN plate reader.

### Immunohistochemistry

The piglets were sacrificed by CO_2_ inhalation. The livers were removed and fixed in 4% PFA for 24 h under deep anesthesia. The fixed livers embedded in paraffin and cut into 5-µm sections with a sliding microtome and mounted on glass slides. The sections were dried at 60 °C for overnight and deparaffinized with xylene and graded ethanol. Antigen retrieval was performed by boiling the sections in 10 mM citric acid buffer (pH = 6) at 120 °C for 10 min. Non-specific binding was blocked with 10% goat serum (BOSTRE, Wuhan, China) for 1 h. After blocking, a primary antibody against pig C3 (1:50, Cloud-Clone Corp, Houston, USA) dissolved in 10% goat serum was added to the slides, which were then incubated overnight at 4 °C. Subsequently, a rabbit secondary antibody (1:5,000, Santa Cruz Biotechnology, Santa Cruz, USA) dissolved in 10% goat serum was added to the slides and incubated for 1 h at room temperature, followed by blocking of endogenous peroxidase activity with 0.3% H_2_O_2_ in methanol for 10 min. Visualization was performed using a Vectastain ABC Elite kit (Vector Laboratories, Burlingame, USA). The slides were counterstained with 3,3-diaminobenzidine. Images were taken using a Digital Sight DS-Ri1 camera attached to a Nikon Eclipse 80i microscope.

### Off-target analysis

Putative off-target sites were predicted by online software (MIT CRISPR Design Tool: http://crispr.mit.edu). The genomic regions flanking the OTSs were amplified using genomic DNA of *C3* KO piglets and WT controls and sequenced. Variants were identified by sequence alignment. Indels located around the sgRNA1 target site were considered to be NHEJ-mediated modifications. Primers for OTSs were listed in Supplementary Table S1.

### Statistical methods

Data were expressed as the mean ± SE. *P* values were determined by using student’s t-tests and ANOVA for all quantifications. *P* values < 0.05 were regarded as statistically significant.

## Electronic supplementary material

Supplementary Information
