# Supplementary material for: Generation of complement protein C3 deficient pigs by CRISPR/Cas9-mediated gene targeting
Source: Sci Rep. 2017 Jul 10;7:5009. doi: 10.1038/s41598-017-05400-2 (PMC5503937; doi:10.1038/s41598-017-05400-2)
Supplement: Supplementary file 1 — Supplementary Information [file 41598_2017_5400_MOESM1_ESM.pdf]

Generation of complement protein C3 deficient pigs by CRISPR/Cas9-mediated gene targeting

Wei Zhang[1,*], Guan Wang[1,*], Ying Wang [1,*], Yong Jin[1], Lihua Zhao[1], Qiang Xiong[1], Lining Zhang[1], Lisha Mou[2], Rongfeng Li[1], Haiyuan Yang[1] & Yifan Dai[1]

OTS	Forward primer 5'→3'	Reverse primer 5'→3'	Size of amplicon (bp)	Annealing Temperature (°C)
OTS1	AGGGAGCAGTATTGTTGTT	CGAGTGATTGCCTTGATT	382	53
OTS2	GCCTCCACCTCCATCAAC	CCGTGTCTCCACCACAAA	677	59
OTS3	CATTTACTCCTTGCGTGGTC	CTCGGGACTGTGGTTTCC	645	59
OTS4	GTCTGTGGCTCTTCTTCTG	CATCTGTCCTGCCTTGTA	476	56
OTS5	GCGACCAAGGGACAGCATTA	GGTAACCAGACCCCTGAGCCAGGGT	623	65
OTS6	ATCTTTAACTCCCAACAGT	CCTAGCGAATCATTTCTT	497	50
OTS7	TCTGGGCGACTTCAACGC	AAATGGTCACTGATGGCAAGG	579	59
OTS8	CAGGAAGGGCTGCCAGAGGT	CGTGGTGGGCTTGGGAAAGG	361	64
OTS9	TCTGTGACTATCTCCCAGTT	TTCCCTTAGCAAATACCTT	390	53
OTS10	TGGGACCACAAGCATCAG	GCTACTCACTTCCTCCTTTCC	395	59
OTS11	CTTGCCCATTGCTTAGAACA	TGACAGGGAAGGACGGTGAT	543	58
OTS12	TCTGAGCCTTAGTTTCTTCTGC	GCTTTCTGGATGGGTCTGTT	622	58
OTS13	TTCTCGGCTTCTGCATTTGT	CCATCCCATGAACGGTCTTA	524	56
OTS14	AGTGCGGTAGGGTCTGTA	CTTAGGTGCAATAGGATG	517	55
OTS15	AATAGTAGAGGGTCTTCTGTCTG	AGTAGGATAATCTTCTGAGGC	384	58
OTS16	GGGAACGAGGAAATGGTC	GGGCAAGAAGGGATAACTG	425	57
OTS17	GCACCTCAACTATTATCT	TAAACCACTTGTCGTCCC	483	53
OTS18	ATGGTCCATGTTGAGAAA	AAGGCAGTATCAGTGTAT	408	50
OTS19	TTCATTAGTCTCATATCCGTAG	GGTTCCATTTCAGGCAGT	315	55
OTS20	GACGAAATCATTGTATCTGCT	GGACTCTTGCCTTTGTGG	574	55
OTS21	ATCAATTCCCTGGCTTTC	ATCTTGGAGTTGGCTTCT	357	53
OTS22	CAGAGGTGAGGGAGGAGAAAGG	CAAGCAGGGTCAGCAGACAG	590	63
OTS23	TCCTGGGATGCTTGTTCG	GGCTGCGGCTGTACTTTT	324	57
OTS24	GGCATCTGTCTGGTAAGGG	AGAAACTGGGCACGCTGA	584	58
OTS25	TTTTGATCCCAGAAACATTG	GACAACACCCTCGCCCTA	460	55
OTS26	GCTGGCATGTGACTTTCT	TGGAGGGTCTGCTTGTAG	436	56
OTS27	AGGTGAGGTCAGAATGCAGGAG	AGTGGCGGGTGGGAGAAA	367	61

Table S1 Primers for off-target sites detection.

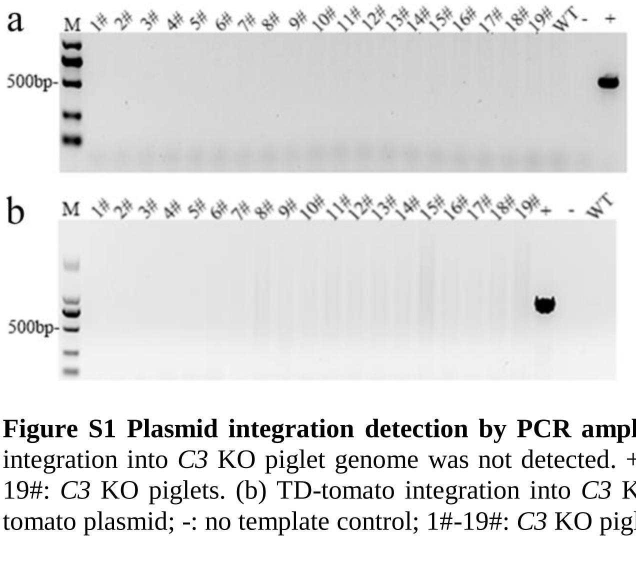

Figure S1 Plasmid integration detection by PCR amplification. (a) Cas9-sgRNA targeting vector integration into *C3* KO piglet genome was not detected. +: pX330 vector; -: no template control; 1#-19#: *C3* KO piglets. (b) TD-tomato integration into *C3* KO piglet genome was not detected. +: TD-tomato plasmid; -: no template control; 1#-19#: *C3* KO piglets.

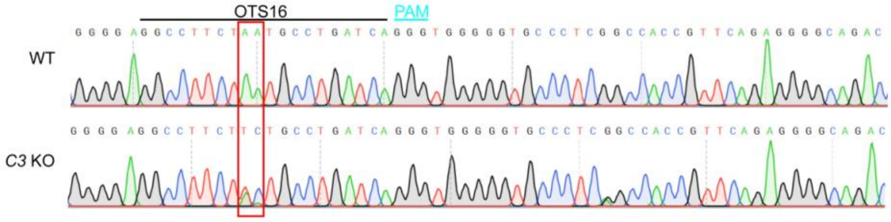

Figure S2 Sequences around the OTS16.

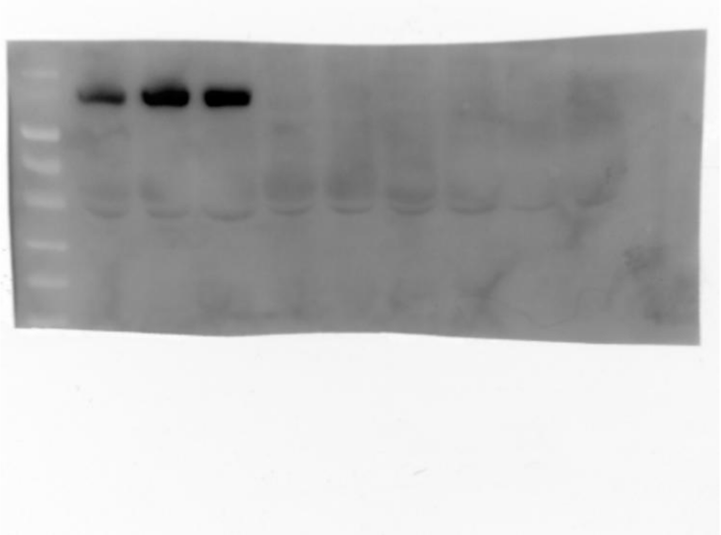

Figure S3 The full-length blots of serum C3α.

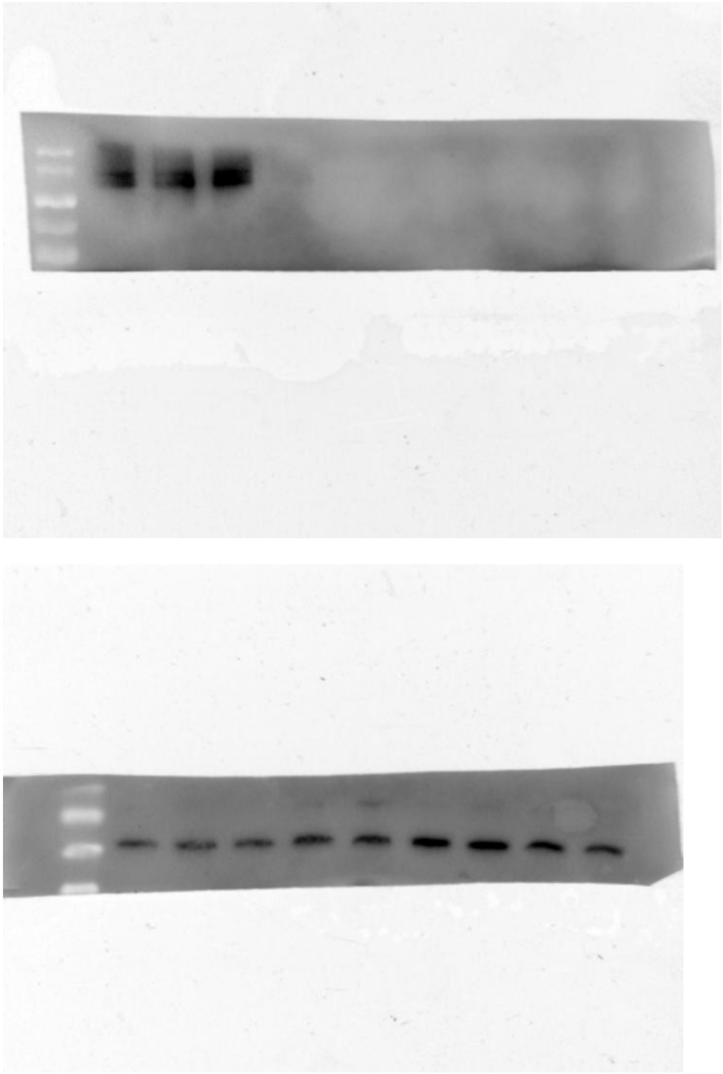

Figure S4 The full-length blots of liver C3α (up) and GAPDH (down)

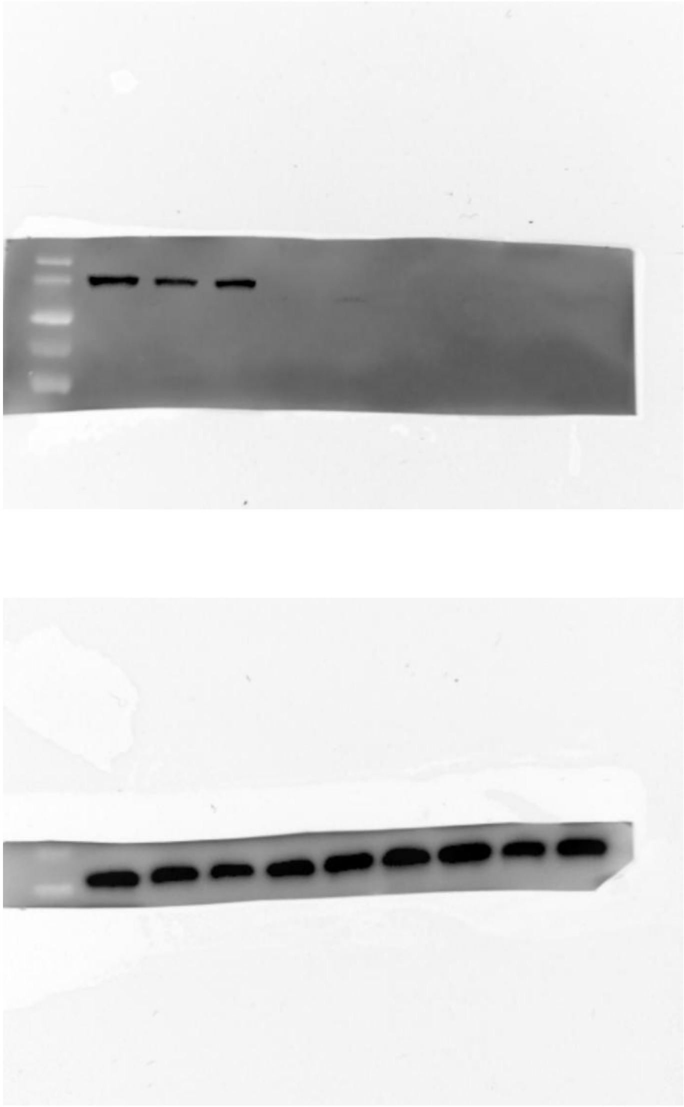

Figure S5 The full-length blots of lung C3α (up) and GAPDH (down)

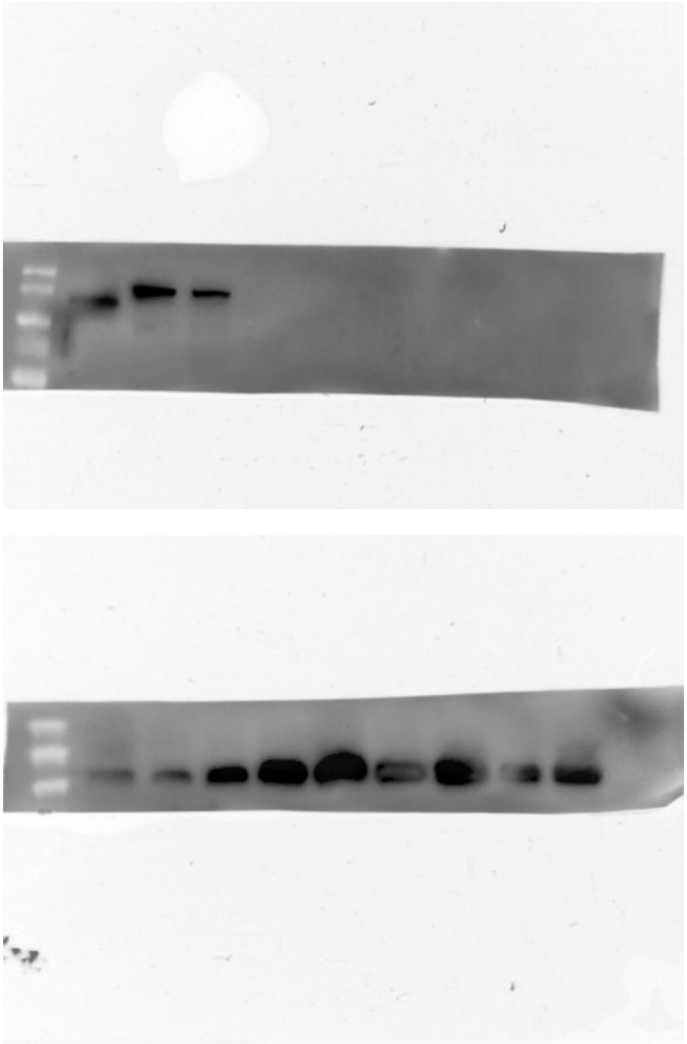

Figure S6 The full-length blots of kidney C3α (up) and GAPDH (down)

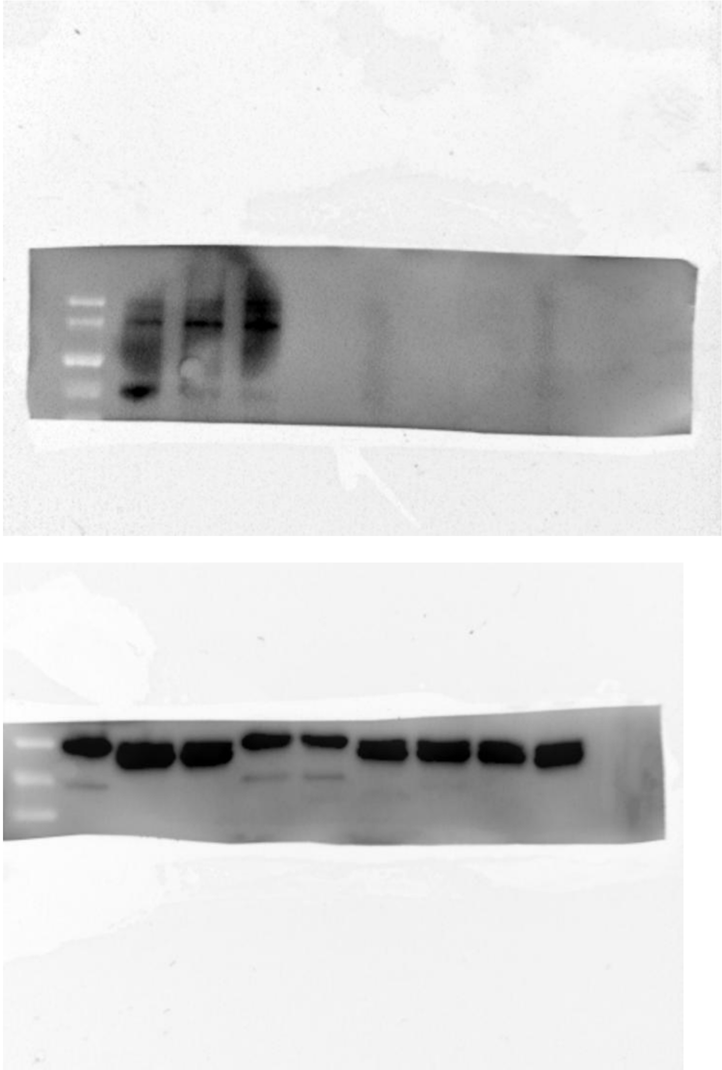

Figure S7 The full-length blots of spleen C3α (up) and GAPDH (down)
